# Data-hugging shields proprietary AI models from research that could disprove them

**DOI:** 10.1038/s44387-026-00094-2

**Published:** 2026-04-17

**Authors:** Anish Karpurapu, Zhicheng Guo, Xiao Hu, Cynthia Rudin

**Affiliations:** 1 Medical Scientist Training Program, Duke University School of Medicine, Durham, NC USA; 2 Department of Computer Science, Duke University, Durham, NC USA; 3 Nell Hodgson Woodruff School of Nursing, Emory University, Atlanta, GA USA

**Keywords:** Cardiology, Computational biology and bioinformatics, Health care, Medical research

## Abstract

“Data hugging” blocks independent verification of medical AI. Apple claims age estimation with a mean absolute error of 2.9 years using photoplethysmographic (PPG) signals. Given PPG’s noise, such accuracy is questionable, raising concerns about other tech companies’ claims. Using UK Biobank data, we find this accuracy unreplicable, achieving results only marginally better than predicting mean age. We advocate for curated public benchmark datasets and evaluation platforms to protect the public from unverifiable claims.

## Introduction

“Water, water everywhere, nor any drop to drink” from *The Rime of the Ancient Mariner*, by Samuel Taylor Coleridge, nicely describes almost every academic AI health researcher’s challenges with access to data.

Medical AI has always promised to revolutionize healthcare, from early disease detection to personalized treatment, but its success hinges on robust evidence and public trust. Yet today, many cutting-edge health AI models are developed behind closed doors on proprietary datasets, precluding independent verification. Data and code sharing is remarkably scarce in medical AI literature: a recent systematic review of 1342 studies of AI in critical care found that 85% of studies did not make their datasets available, and 87% of studies did not provide relevant code^1^. Such widespread absence of transparency and reproducibility impedes external validation, hampers cumulative scientific progress, and, as we show here, leads us to believe we are safer than we are.

Several high-profile failures of prominent AI algorithms have already surfaced: Epic’s widely used sepsis prediction algorithm was shown to have poor performance on external retrospective validation, after it had already been deployed in hospitals across the United States^2^. Likewise, Philips recently issued a Class I software correction for its outpatient telemetry monitoring service when the algorithm failed to transmit ECG alerts for atrial fibrillation and ventricular tachycardia, contributing to two patient deaths and 109 injuries^3^. Perhaps these were anomalies; however, could this happen more often for applications where we do not see immediate effects, and we just do not know about it? How broad a scale do these errors reach? One prominent case arises from the recent revolution in heart monitoring enabled by smartwatches. Wearable-based detection of heart issues presents a major opportunity, as many people remain unaware of cardiovascular problems until serious events occur. Atrial fibrillation (AFib), in particular, is a common yet often undiagnosed condition that significantly increases the risk of stroke^4^. Apple claims to detect AFib from smartwatch-derived photoplethysmographic (PPG) signals with exceptionally high accuracy^5^, offering the potential to improve outcomes for millions through early intervention. However, the lack of access to Apple’s dataset, or any comparable large public dataset, prevents independent researchers from verifying these claims. Should such claims from entities like Apple be accepted without independent scrutiny? They performed experiments on a proprietary large-scale dataset and reported extensive results to the FDA^6^. However, a previous independent evaluation on the Apple Watch showed that the detection algorithm could not classify 27.9% of ECG signals that were provided to it^7^. More disconcertingly, *other* claims presented by Apple strain credulity. Specifically, Apple recently presented results indicating that they could predict a person’s biological age along with other demographic features purely from PPG signals collected via Apple Watch, with an average error of roughly *three years* for age prediction^8^, while the rest of the field was achieving error around seven to ten years^9–20^. Subsequent Apple papers reported even lower errors (2.89 years using PPG alone and 2.46 years with behavioral features^21^; ~2.4 years in a self-reported healthy cohort and ~3.2 years in the general cohort^22^). However, these results still lack routine independent replication because the Apple Heart and Movement Study dataset is not publicly available; releasing code and synthetic data cannot substitute for external validation on real, independent datasets^22^. These escalating, state-of-the-art claims, produced on the same inaccessible Apple Heart and Movement Study dataset, further highlight the widening chasm between proprietary results and what is verifiable by the broader scientific community.

Any of these results, if validated, would be remarkable. They would imply that age information, accurate to within approximately three years, could be collected with light beamed into surface vessels on one’s wrist over a short period of time, independent of genetics or lifestyle factors such as exercise or smoking history—factors that typically create discrepancies between vascular age measures and chronological age.

While not entirely discounting biological plausibility, several factors render these claims challenging to accept at face value. First, measurements of vascular aging generally are not able to pinpoint age that precisely^17^; even epigenetic measures of aging are only able to estimate age to about 3.6 years^23^. Second, neither of Apple’s papers used a single external or public dataset. The authors’ models are not public, so there is no way to test model performance on external datasets, and their data remains proprietary, preventing any validation of their findings. Third, their claims lack interpretability, and as such, the paper does not provide any additional scientific insight or benefit to the scientific community that could be used to verify the claims or inform future research.

**Fig. 1:**
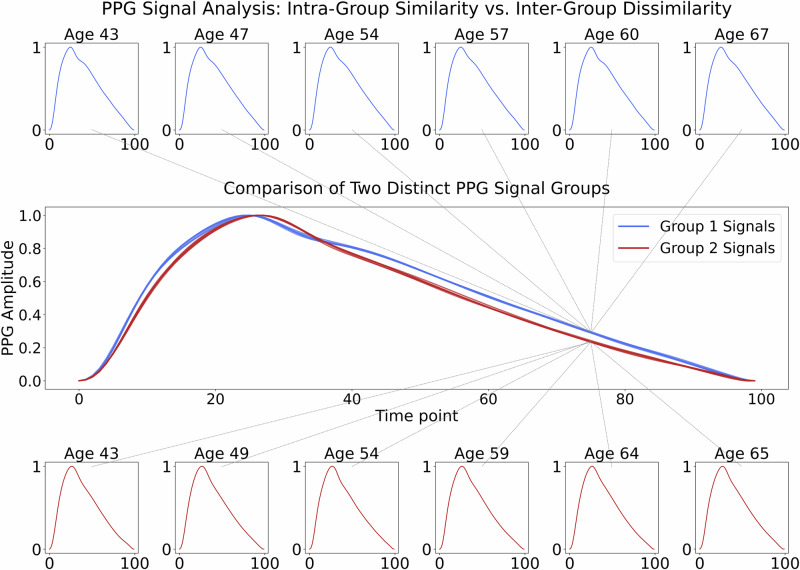
Two distinct PPG signal morphologies (identified through cross-bucket similarity analysis, where similar signals are placed into the same group) are almost identical across multiple ages. The main panel shows two representative groups of PPG signals (Group 1: blue; Group 2: red), each containing six signals from *different* 5-year age buckets spanning 40–70 years. These results demonstrate high similarity within each group and clear dissimilarity between groups, illustrating consistent morphology despite age variations. Inset panels display individual signals with participant ages (top: Group 1; bottom: Group 2).

**Fig. 2:**
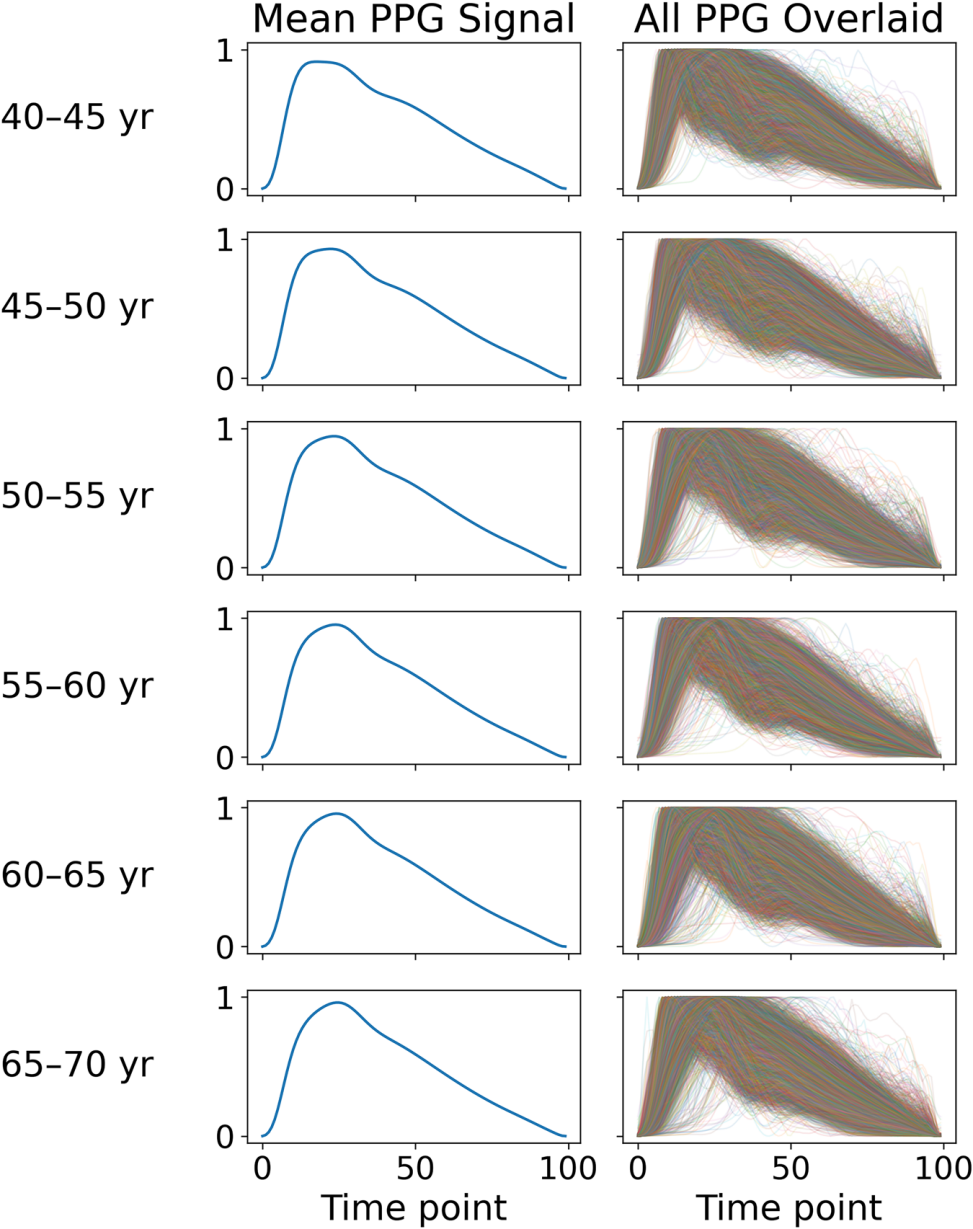
Paired plots show the mean PPG for each 5-year age group, followed by an overlay of all PPGs for each age group. This figure illustrates substantial variation within each age group.

If we could determine whether Apple’s claim on age prediction is replicable, it would help determine whether its accompanying results on atrial fibrillation (which is a more clinically significant condition to detect) are valid. In other words, *by testing claims that are plausible to test using public data, we will either verify an amazing discovery or cast doubt on related (important) results that we have insufficient data to verify*.

Thus, in this work, we used public PPG data to try to evaluate Apple’s reported findings on age. If Apple’s results are replicable, we would expect to see at least some predictability in age. *But we did not find much improvement beyond just predicting the mean population age*. Our analysis, carried out using a deep neural network comparable to the one used in Apple’s studies^8,21^, both trained on the UK Biobank^24^, yields results more consistent with physiological understanding than Apple’s reported outcomes. Specifically, *we observed substantial variability in PPG signals among individuals of identical chronological ages* (see Fig. 1) and *high similarity in some PPG signals across different chronological age groups* (also see Fig. 2, Supplementary Information Fig. S1, and Supplementary Information Fig. S2), which indicates low predictability of age from PPG morphological features alone. Our results indicate that there is a fairly low ceiling for predictive accuracy in biological age estimation. They suggest that Apple’s claims may not be biologically plausible or generalizable without substantial additional covariates or interpretive context.

**Fig. 3:**
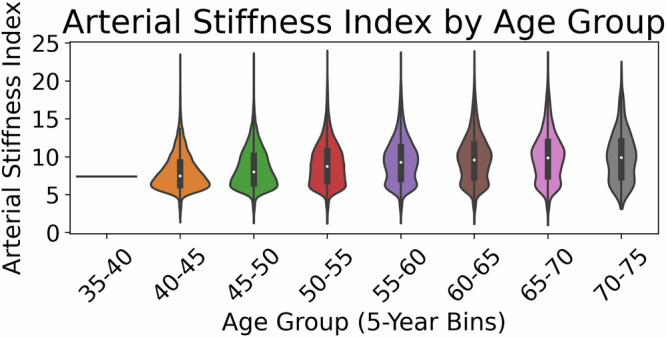
Violin plot for arterial stiffness index (ASI) plotted versus 5-year age groups in the UK Biobank cohort. Bars show the Q1–Q3 range, and dots show the median. For this visualization, data points falling below Q1 − 3*IQR or above Q3 + 3*IQR were considered outliers and removed. The within-age-group variation is always large.

**Fig. 4:**
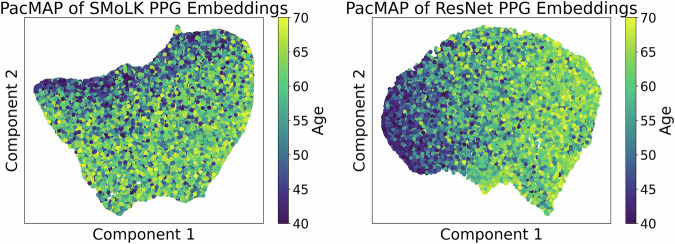
PaCMAP projections of learned embeddings from a randomly selected representative model in fivefold cross-validation. *Left:* PaCMAP of SMoLK model embeddings on validation fold. *Right:* PaCMAP of ResNet model embeddings on validation fold. Age does not dominate the learned representations.

Our results cast doubt on some of the largest-scale and most important types of results in AI health, which is problematic because atrial fibrillation detection is important. We place the blame for this circumstance purely on “data hugging,” i.e., not sharing data. There is simply no high-quality, large, public PPG dataset, nor a public testing platform for PPG, from any source on-par with the Apple study. If there were, we could test Apple’s results on atrial fibrillation directly.

While gathering and maintaining high-quality data is challenging^25^, we claim that constructing and maintaining these datasets and testing platforms is completely within the realm of possibility and has precedent. The National Institute of Standards and Technology (NIST) has been running facial recognition vendor tests, as well as speaker recognition vendor tests, since 1996^26–29^. This approach could readily extend to health data. If anyone were to devote the same effort to specific health data systems as NIST does to facial recognition and speaker recognition systems, it could lead to a measurable and almost immediate improvement in human health, with many lives saved.

Below, we first discuss Apple’s study, then examine the problems with public PPG data to contextualize the disparity between what companies possess and what the public can access. We then present our effort to predict age using public PPG data, discuss the implications of our results on age estimation, and propose remedies that were briefly outlined above. *In short, our position is that data hugging is becoming increasingly problematic due to the increase in widespread proprietary models, and that there is a practical remedy, which is the development of a few, highly curated public benchmark datasets and evaluation platforms*.

## Apple’s study: do we trust the results?

A research paper by Apple recently presented a striking advance: a foundation model trained on a large dataset of PPG and electrocardiogram (ECG) data from the Apple Heart and Movement Study^8^. The study enrolled approximately 141,000 participants aged 18 years and older, with no upper age limit, who contributed continuous wrist-based PPG and ECG recordings collected via Apple Watch over a period of up to 3 years. Apple’s self-supervised foundation model, trained on this proprietary dataset, reportedly encodes participant characteristics effectively enough that researchers can predict demographics with very high accuracy using linear models on its features. For example, using PPG data, the model was able to distinguish older vs. younger individuals (above vs. below 50) with an AUC of 0.976. Moreover, in the regression task, the model could predict a patient’s exact age with a mean absolute error of only 3.2 years. These results were followed by an even more striking claim from a subsequent Apple study, which reported a mean absolute error of 2.46 years by combining sensor and behavioral data^21^.

A closely related follow-up study, Miller et al. (2025), introduces a wrist-PPG “aging clock” (PpgAge) and reports associations between age-gap statistics and cardiometabolic disease, behaviors (smoking, exercise, sleep), and longitudinal physiological events^22^. However, neither the models nor the underlying data are publicly available, leaving the reported performance unvalidated on independent datasets.

Age estimation and atrial fibrillation detection are totally different problems. We know we cannot externally validate Apple’s claims with respect to the detection of atrial fibrillation since we do not know what data were omitted. Atrial fibrillation is easy to detect if the data are clean^30,31^ however, the data are essentially never clean. One way to find almost perfect accuracy in atrial fibrillation detection is to report accuracy after omitting all of the noisy signals, which could be a sizable fraction of the data. But omitting much of the data negates the point of using the device in the first place. In contrast, age does not rapidly change over time like atrial fibrillation signals, and the reported age-prediction error of roughly 2–3 years is substantially tighter than previously demonstrated on public datasets. While potential confounders such as medication use or disease status may contribute to age prediction, these factors alone are unlikely to explain near-perfect accuracy, and possibilities like inadvertent data leakage or methodological errors cannot be independently assessed without access to the data or model. These considerations make age prediction a particularly informative case for assessing replicability.

## Public data on PPG and age is woefully insufficient

Despite the unprecedented potential for heart monitoring to save lives through early disease detection, publicly accessible datasets for PPG signals, particularly those annotated with critical labels like age, remain limited in size, scope, and detail. Currently, the largest publicly accessible dataset is the UK Biobank, consisting of finger-based PPG recordings for ∼170,000 patients, roughly between the ages of 40 and 70^24,32^ at the first assessment visit. Each waveform consists of a single pulse of 100 points collected after the waveform stabilizes over 10–15 s. Other public PPG datasets are typically orders of magnitude smaller. For example, the MIMIC database consists of on the order of 10,000 critically ill ICU patients rather than a general population cohort^33^. Other public datasets are often drawn from specialized contexts, such as sleep studies or physical activity trials, and include many fewer patients. Some notable datasets include the WESAD dataset, consisting of PPG signals from 15 participants undergoing a lab study of stress detection^34^, PPG3 DaLiA with continuous physiological PPG recordings from 15 participants during daily activities^35^, Stanford Wearables Dataset consisting of about 50 patients monitored longitudinally over several months^31,36,37^, VitalDB consisting of about 6000 PPGs recorded during surgical cases^38^, MESA with about 2,000 patients who underwent a sleep study and had PPG signals recorded throughout^39^, and the nuMoM2b study, recording overnight PPG signals during a sleep study for over 3000 moms in pregnancy^40^. Only recently have initiatives like PaPaGei begun to aggregate existing public datasets, achieving about 13,500 patients from publicly available datasets for the purpose of training a foundation model^41^. However, these efforts remain nascent, and widespread adoption is still limited.

All publicly available datasets have critical limitations, including short recording lengths (typically 30-s clips), preventing long-term analyses, inadequate sample sizes (sometimes as few as 5 patients), and incomplete covariate information, such as smoking status or body mass index. Even the UK Biobank, currently one of the most comprehensive public health datasets, presents substantial challenges for training deep learning models, including cost, long wait times for access, inability to download individual data directly, and infrastructure issues on the DNANexus Platform. Operationally, utilizing the UK Biobank via DNANexus constrains researchers to work within its specific cloud infrastructure, necessitating careful resource management to avoid job termination during computationally intensive tasks like deep learning model training and to manage usage-based costs. These costs are incurred for compute utilization, storage of data and models, and data egress. For example, running an on-demand instance with 144 GiB of memory for 48 hours (mem1_ssd1_v2_x72) would cost £94 on the platform, in addition to a £3000 3-year Tier 1 access fee. The requirement to perform computations exclusively within this environment, along with the high costs, influences the structure and feasibility of large-scale, iterative deep learning experiments.

Lack of public data means: (1) potential biases go unchecked until deployment [see the dermatology study of Ibrahim et al. ^42^], including those due to the goal of misleading readers [see the critique of Kan-Tor et al. ^43^, about an IVF study] (2) underrepresented groups may be excluded [e.g., “health data poverty,” ^44^], (3) errors in coding or reporting go unchecked^45^, (4) generalizable knowledge or insight about the medical condition itself cannot be gained—the revolution in health data science is stalled precisely because of these issues.

## Predicting age using the UK Biobank disagrees with Apple’s findings

We aimed to replicate Apple’s findings using the UK Biobank dataset. The dataset provides PPG measurements alongside a wealth of other clinical data. We focused on data from each patient’s first assessment visit conducted between 2006 and 2010, which includes finger PPG recordings from 170,624 patients.

### Analysis 1: arterial stiffness index, a known age estimator

We first studied how the arterial stiffness index varied as a function of age. The arterial stiffness index (ASI) is an established biomarker for vascular aging and is strongly linked to cardiovascular risk factors^46,47^. It quantifies the ability of arterial walls to expand and contract in response to blood flow pulses. Diminished arterial compliance, often associated with aging, can be quantified directly from PPG signals using the formula ASI $=\frac{\,{\rm{height}}}{{\rm{peak-to-peak\; distance}}}$, where peak-to-peak distance is defined as the time interval between the initial systolic peak and the subsequent (or reflected) peak in the pulse waveform, effectively serving as a surrogate for the pulse wave transit time through a patient’s arterial tree. In our analysis, we see a trend where ASI increases as patients age. Figure 3 and Supplementary Information Fig. S3 present summary statistics for the distribution of the arterial stiffness index for the UK Biobank. For instance, the median arterial stiffness index rises from approximately 7.3 m/s at age 40 to nearly 9.9 m/s at age 70. However, the substantial inter-quartile range observed within each age group indicates that age alone does not fully explain the variance seen in arterial characteristics. The notable spread in these values points to the existence of additional factors—such as lifestyle choices and comorbidities—that may significantly modulate arterial stiffness. For example, the third quartile ASI for age 40 at 8.8 m/s is much larger than the median ASI for age 50 at 8.4 m/s. The implication is that ASI, one of the most well-defined and reliably calculated features from a patient’s PPG^48^, is not sufficient to determine a patient’s age to within 3.2 years. To quantify this ceiling directly, we evaluated an ASI-only baseline for predicting chronological age using fivefold cross-validation, fitting (i) a univariate linear model and (ii) a spline-based generalized additive model (GAM) within each training fold and evaluating on the held-out fold. The linear baseline achieved an average MAE of 6.85 years, while the GAM provided a small improvement with an MAE of 6.78 years. Both are only slightly better than predicting the mean age (Table 1). These results provide a reference point for how much age signal is captured by a single, explicitly defined vascular feature derived from PPG.

**Table 1:** Performance comparison for predicting the mean

Model	MSE (years^2^)	MAE (years)	Spearman *ρ*
SMoLK	51.77 ± 0.32	5.97 ± 0.02	0.44 ± 0.00
ResNet	42.47 ± 0.60	5.24 ± 0.05	0.57 ± 0.00
ASI-only (linear)	64.88 ± 0.46	6.85 ± 0.01	0.20 ± 0.00
ASI-only (GAM spline)	63.58 ± 0.12	6.78 ± 0.01	0.20 ± 0.00
Mean baseline	66.52 ± 0.35	6.98 ± 0.02	N/A

Mean squared error (MSE), mean absolute error (MAE), and Spearman’s Rho (*ρ*) are reported as mean ± standard deviation over fivefold. The mean baseline predicts the average age from the training set for all validation samples.

### Analysis 2: deep learning for age prediction

It is possible that Apple’s foundation models^8,21^ learned new features or a mix of features to more precisely predict a patient’s age. To explore this possibility, we conducted experiments using two distinct deep learning architectures: SMoLK^49^, a lightweight yet powerful model suitable for wearable devices, and a ResNet-based model^50^, which is commonly used in PPG-related tasks, including classification^31,51^ and as the backbone for PPG foundation models^52^. Our guiding hypothesis was that a sophisticated architecture might be able to learn latent features beyond ASI to predict a patient’s age from PPG data. Our results, however, were modest at best compared to Apple’s results, suggesting that even sophisticated deep learning architectures face significant challenges in accurately predicting age from PPG data in isolation.

We show the results of our models in Table 1. These performance metrics indicate that while the models capture some signal related to age, likely related to vascular aging, the magnitude of confounding factors inherent in the dataset severely limits the models’ ability to predict age with greater precision than within half a decade. The details of model training and evaluation are described in the Supplementary Information.

To further understand the nature of the features learned by our models, we employed PaCMAP (Pairwise Controlled Manifold Approximation Projection), a dimensionality reduction technique designed to preserve both local and global structures in high-dimensional datasets^53^. PaCMAP was used to project the embeddings derived from the trained ResNet and SMoLK models into a two-dimensional space, thereby facilitating visual inspection of how well the age-related signal was separated by these models (Fig. 4). Our PaCMAP visualizations reveal that, although one of the primary axes in the embedding space exhibits a discernible gradient corresponding to age, the overall distribution remains highly overlapped. Thus, while age does have an observable influence on the PPG signals, it does not dominate the learned representations.

Instead, the embeddings may capture the influence of other factors—likely including lifestyle aspects, genetic background, and additional health markers—which collectively introduce substantial confounders into the age prediction latent space. For instance, we know that hypertension and elevated triglyceride levels tend to increase with age and contribute to arterial stiffness and a change in vascular profile^54^, but these do not increase at the same *rate* for everyone.

Thus, as with all previous age indices, we are able to extract some features that correlate with age, but none are able to predict it to within an average of 3 years, or even 5 years.

## Connecting our results with Apple’s analysis as a reality check

There are several differences between our study and Apple’s, some stemming from limitations of public data. First, our empirical analysis relies on the UK Biobank dataset, which, while large, has specific characteristics: the PPG signals are recorded from the finger at rest for a short duration, unlike the continuous wrist-based PPG in the Apple study. This difference in sensor placement (finger versus wrist) introduces variability in signal characteristics as finger PPG often provides a higher fidelity signal and allows for more stable measurements^55,56^. Furthermore, the UK Biobank cohort primarily includes individuals between the ages of 40 and 70, while the Apple Heart and Movement Study consisted of patients as young as 18 years old with no upper limit. While we explored several state-of-the-art deep learning architectures (e.g., SMoLK and ResNet), it remains possible that other model architectures or more extensive tuning could yield different results. However, we do not believe these differences alone would allow for the substantially superior age estimation accuracy reported by Apple.

As discussed, Apple did not provide any interpretation of their results as to what features in the PPG signal the model may be leveraging. Our UK Biobank findings raise important questions about whether Apple’s model found a true biological signal of aging or if it capitalized on idiosyncrasies of the Apple Heart and Movement Study. In the absence of Apple releasing its model, data, or any insight into what its model has detected, we cannot dissect its features. But the broader point is that closed models invite skepticism because our results disagree, and alternate explanations cannot be ruled out.

In summary, our analyses using openly available data serve as a reality check on Apple’s claims. This reinforces the need for external model validation. Without that, even well-intentioned studies risk painting an overly optimistic picture. For the field of medical AI to progress and for the public to benefit, we must bridge the gap between isolated, privatized results and generalizable science.

## Potential remedies

The challenges previously identified are not insurmountable, even if we cannot expect a sea change in transparency anytime soon. We propose several concrete, realistic remedies to foster a more open, trustworthy, and efficient ecosystem for medical AI, as well as some more general remedies.

### A few comprehensive open-source benchmark datasets and independent evaluation platforms can have a huge impact

A crucial remedy lies in the strategic development and maintenance of a few high-quality, large-scale, open-source benchmark datasets along with independent evaluation platforms, focusing on specific high-impact health domains. The selection criteria for these domains should prioritize conditions that (1) represent common deadly diseases; (2) allow for meaningful prediction or early detection using data collected before severe disease manifestation; and (3) involve data types that are widely collected and are not difficult to collect, making large-scale dataset creation feasible.

Let us discuss several prime examples meeting these criteria, starting with cardiovascular monitoring for heart arrhythmias such as atrial fibrillation. As discussed, while wearables collect vast data with potential for early arrhythmia detection, proprietary control of these data blocks the independent validation needed to trust life-altering claims – a dangerous paradox given the stakes. A dataset could be easily collected with a concerted effort from a large group of individuals at risk for heart arrhythmia.

A second prime example is medical imaging for cancer screening. Ubiquitous mammography data offers a clear path to AI contributions, yet frustrating access limitations stall independent validation and broader progress. For instance, a recent breakthrough in AI-mammography shows that breast cancer can now be predicted up to 5 years in advance from subtle asymmetries between the patients’ left and right sides^57^; however, little can be done to advance this technology to the clinic without access to large datasets, despite the fact that doing so could potentially prevent numerous cancers and save lives. Mammography data are collected in virtually every large medical center and numerous additional clinics, but they are not generally available to academic researchers.

There are several other examples. Digital pathology requires large datasets to train robust models for analyzing whole-slide images for cancer diagnosis and grading; yet, access is often institutionally siloed. Similarly, developing and validating generalizable, fair models for predicting chronic disease onset or progression using longitudinal electronic health records demands large, diverse EHR datasets. Dermatology could benefit from diverse benchmark datasets to improve AI-based skin cancer detection. Furthermore, standardized public validation datasets are scarce for many at-home monitoring applications, like cuffless blood pressure estimation, sleep staging from wearables, and diabetic foot ulcer imaging, despite the feasibility of frequent and inexpensive data collection.

These benchmark datasets must be accompanied by robust, independent evaluation platforms. There is a clear precedent for empowering neutral agencies like the National Institute of Standards and Technology (NIST) to evaluate high-stakes AI systems. A similar initiative in medical AI, potentially led by NIST, the FDA, or a trusted independent consortium such as the proposed Health AI Assurance Lab^58^, could establish transparent and standardized evaluations with both overall and demographic-specific performance metrics.

Importantly, these platforms and their associated datasets would require investments for regular updates and versioning to reflect evolving clinical practice and prevent overfitting to static benchmarks^59^. Aligning these evaluation standards with regulatory needs for post-market surveillance and managing algorithm changes would further enhance their value. While establishing such infrastructure requires investment, the potential return in terms of accelerated innovation and improved human health could be immense.

### Personalized algorithm evaluations can help users choose between AI-health products

While the evaluations discussed above would lead to major health innovations, they do not address patient-specific decisions, for instance, whether *you* should buy an Apple Watch or a Fitbit. The answer would depend on which brand’s algorithm works better for you. Currently, there is no way for consumers to evaluate that information. It could be incredibly helpful to consumers of AI-health algorithms to be able to upload personal data into an evaluation platform to determine which AI-health product is best for them. In doing this, it is important to protect individual medical data uploaded into such an evaluation platform – no customer’s data should be stored or used for any other purpose than to evaluate algorithms so users can choose between them.

### The paradigm of data ownership and sharing needs to change

The current view of data as investigator-owned should be reconsidered. Patients and, by extension, the general public have a stake in the data they contribute. Datasets should generally be treated as shared assets serving broader societal interests, not just proprietary assets for investigators with privileged patient access.

This paradigm shift may require examining the role of Institutional Review Boards (IRBs) in research ethics. One of the IRB’s mandates is to safeguard participants while upholding ethical standards and regulations^60^. Yet a critical question emerges: Is it possible that study participants are treated unethically if *they are never informed that their data will be involved in a non-reproducible study*? Patients should be able to choose to share their de-identified data to enable scientific reproducibility and further studies. It is also possible that study participants and the public are *substantially worse off* when either (1) the study is not reproducible, and the results turn out to be wrong; (2) the data is used by only one research group rather than many. Might it be possible, then, that IRBs can inadvertently perform ethics-washing for data-hugging?

In the United States, this dynamic is compounded by the unintended consequences of privacy regulation. HIPAA was originally intended to prevent private health insurance company malfeasance, but it has since become a barrier to data access for legitimate research. This policy regime allows EHR and PACS vendors to keep their versions of collated and extensive datasets proprietary. As we have noted, these very vendors are often the ones making consequential AI mistakes (e.g., Epic’s sepsis algorithm and Philips’ telemetry failures). The result is a paradox: regulations designed to protect patients now shield the organizations whose algorithms may be putting patients at risk from the external scrutiny that could identify and correct these errors.

IRBs could extend ethics oversight beyond study participants to include all stakeholders, such as funders, taxpayers, and the broader public who may be affected by the study’s outcomes. Specifically, IRBs would require actionable data custodianship plans that treat data as a public resource to help prevent data hugging.

IRBs could reduce the restrictive language from informed consent forms and procedural loopholes that sometimes relieve investigators of the responsibility for meaningful data sharing. Consent forms could also default to permit future secondary use, while distinguishing real privacy risks from administrative hurdles. Lastly, institutions should offer standardized data preparation pipelines and vetted templates that ensure compliance with HIPAA^61^, GDPR^62^, and FAIR principles^63^.

## Conclusion

We are awash in a sea of medical studies, almost none of which we can actually trust. The advancement of trustworthy medical AI is critically hampered by data-hugging practices that shield proprietary models from independent scrutiny. Using Apple’s claim of high-accuracy age prediction from PPG signals as a case study, we demonstrated through analysis of the public UK Biobank dataset that such accuracy appears challenging to replicate with currently available public data and methods, as we achieved results consistent with prior literature (and close to random guessing) rather than the recent Apple claims. This discrepancy underscores the urgent need for mechanisms to validate high-impact health AI models, especially those deployed at scale via consumer wearables. We argue that establishing a few strategic, large-scale, open benchmark datasets and independent evaluation platforms is feasible and essential. We call upon the research community, funding bodies, regulators, and industry stakeholders to prioritize the creation of these vital public resources to foster innovation grounded in verifiable science.

## Supplementary information

Supplementary Information

## Data Availability

This research was conducted using the UK Biobank Resource (Application ID 549696). UK Biobank data are available to bona fide researchers upon application at www.ukbiobank.ac.uk.

## Code availability

The full analysis code (data processing, training, and figure generation) can be found at https://github.com/agk2000/PPG-Age-Reproducibility.
