# Supplementary material for: Data-hugging shields proprietary AI models from research that could disprove them
Source: NPJ Artif Intell. 2026 Apr 17;2(1):45. doi: 10.1038/s44387-026-00094-2 (PMC13090122; doi:10.1038/s44387-026-00094-2)
Supplement: Supplementary file 1 — Supplementary Information [file 44387_2026_94_MOESM1_ESM.pdf]

Supplementary Information

Supplementary Figures

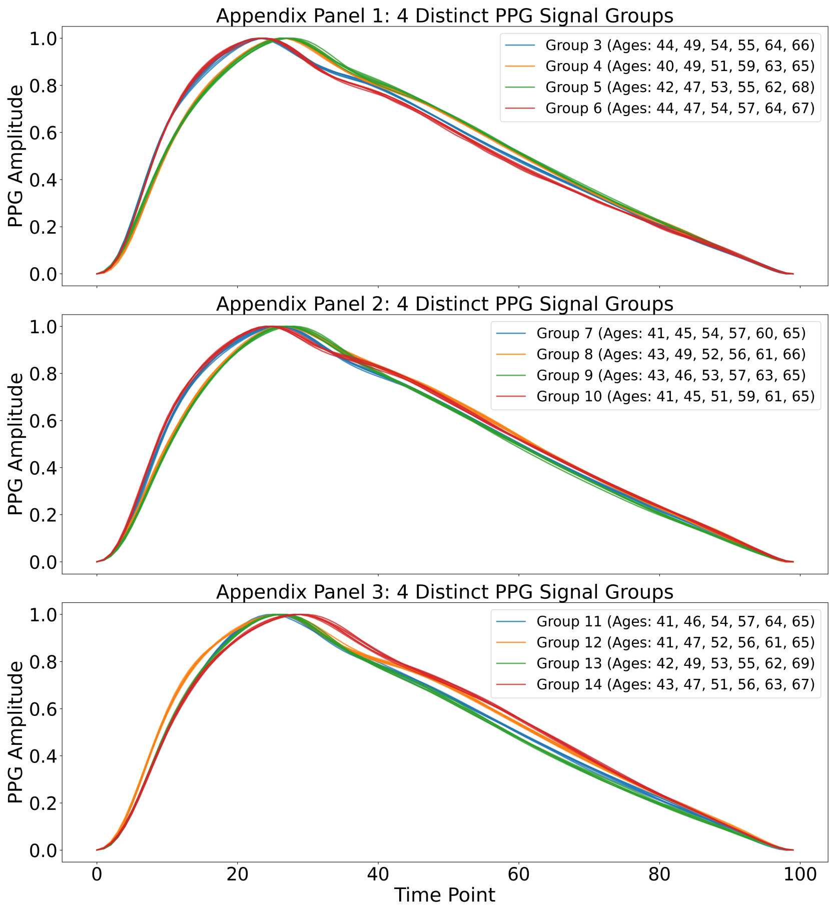

Supplementary Fig. S1 Additional examples of distinct, age-persistent PPG signal morphologies. This figure presents three panels, each showcasing four unique groups of PPG signals (labeled Group 3 onwards, differentiated by color). Each group consists of six signals, including one from each 5-year age bucket (40-70 years), demonstrating high signal similarity within each group, yet across a wide variety of ages. All groups are unique and distinct from those in Figure 3 and from each other.

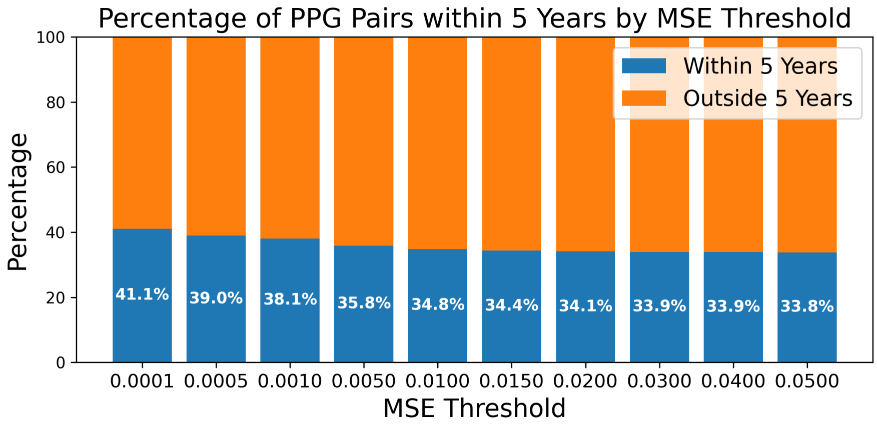

Supplementary Fig. S2 Proportion of PPG signal pairs from the UK Biobank where participants' ages are within 5 years of each other, stratified by Mean Squared Error (MSE) thresholds. Data were generated by sampling 1,000 individuals from each 5-year age bucket (40-70 years). For each MSE threshold indicated on the x-axis, the stacked bar shows the percentage of signal pairs with an MSE less than or equal to that threshold that fall into two categories: participants whose ages are within 5 years of each other (blue), and those whose ages are more than 5 years apart (orange). The consistently high proportion of pairs with age differences greater than 5 years, even at very low MSE values (indicating high PPG similarity), suggests that PPG morphological features alone are weak indicators of close chronological age proximity in this public dataset.

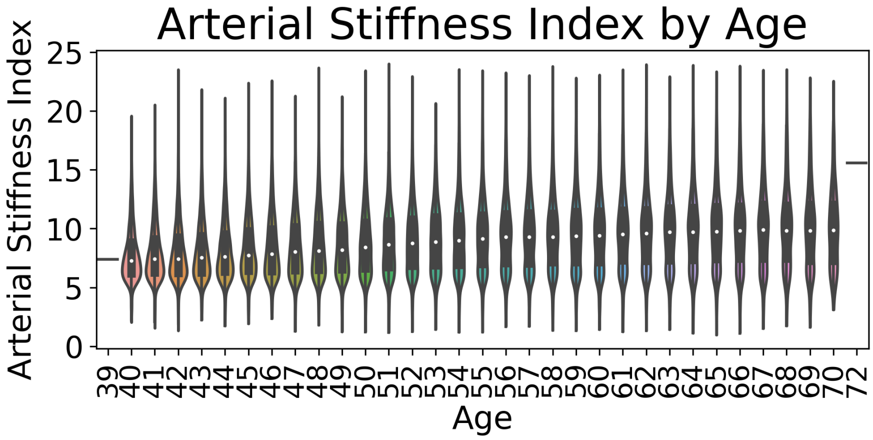

Supplementary Fig. S3 Violin plots for arterial stiffness index (ASI) plotted versus age in the UK Biobank cohort. Bars show Q1-Q3 and dots show the median value. For this visualization, data points falling below Q1 - 3*IQR or above Q3 + 3*IQR were considered outliers and removed. The within-age-group variation is always large.

Model detail

SMoLK is a single-layer sparse neural network that learns a small set of lightweight flexible convolutional kernels designed to capture key features across different time scales from the PPG signal [49]. The SMoLK model was trained for 200 epochs with 384 kernels (kernel sizes of 100, 50, and 20) using learning rates of 0.0001 or 0.001. After each signal was min-max scaled to 0-1, data were trained using 5-fold cross validation on the first instance (corresponding to the initial baseline visit) for the UK Biobank dataset. We also trained a series of one-dimensional ResNets (10 layers deep: one initial 1D convolution, four residual blocks of two convolutions each, and one final fully-connected layer with kernel sizes of 16 and filter-counts of 64, 128, 196, 256, and 320 in the successive residual units) for 40 epochs with learning rates of either 0.0001 or 0.001. Simply predicting the mean age as calculated on the training folds for every patient in the validation fold resulted in an average MSE of 66.52 years^2 and an MAE of 6.98 years across all 5 folds as described in Table 1. We also evaluated ASI-only baselines for predicting age from the arterial stiffness index using the same 5-fold cross-validation split. Within each training fold, we fit a univariate linear regression as well as a spline-based generalized additive model (GAM) and evaluated on the held-out fold. The linear ASI-only baseline achieved an average MSE of 64.88 years^2 and MAE of 6.85 years, while the GAM achieved an average MSE of 63.58 years^2 and MAE of 6.78 years.
